# New Therapies and Immunological Findings in Cutaneous T-Cell Lymphoma

**DOI:** 10.3389/fonc.2018.00198

**Published:** 2018-06-04

**Authors:** Kazuyasu Fujii

**Affiliations:** Department of Dermatology, Kagoshima University Graduate School of Medical and Dental Sciences, Kagoshima, Japan

**Keywords:** cutaneous T-cell lymphoma, mycosis fungoides, Sézary syndrome, primary cutaneous CD30^+^ T-cell lymphoproliferative disorders, adult T-cell leukemia/lymphoma

## Abstract

Primary cutaneous lymphomas comprise a group of lymphatic malignancies that occur primarily in the skin. They represent the second most common form of extranodal non-Hodgkin’s lymphoma and are characterized by heterogeneous clinical, histological, immunological, and molecular features. The most common type is mycosis fungoides and its leukemic variant, Sézary syndrome. Both diseases are considered T-helper cell type 2 (Th2) diseases. Not only the tumor cells but also the tumor microenvironment can promote Th2 differentiation, which is beneficial for the tumor cells because a Th1 environment enhances antitumor immune responses. This Th2-dominant milieu also underlies the infectious susceptibility of the patients. Many components, such as tumor-associated macrophages, cancer-associated fibroblasts, and dendritic cells, as well as humoral factors, such as chemokines and cytokines, establish the tumor microenvironment and can modify tumor cell migration and proliferation. Multiagent chemotherapy often induces immunosuppression, resulting in an increased risk of serious infection and poor tolerance. Therefore, overtreatment should be avoided for these types of lymphomas. Interferons have been shown to increase the time to next treatment to a greater degree than has chemotherapy. The pathogenesis and prognosis of cutaneous T-cell lymphoma (CTCL) differ markedly among the subtypes. In some aggressive subtypes of CTCLs, such as primary cutaneous gamma/delta T-cell lymphoma and primary cutaneous CD8^+^ aggressive epidermotropic cytotoxic T-cell lymphoma, hematopoietic stem cell transplantation should be considered, whereas overtreatment should be avoided with other, favorable subtypes. Therefore, a solid understanding of the pathogenesis and immunological background of cutaneous lymphoma is required to better treat patients who are inflicted with this disease. This review summarizes the current knowledge in the field to attempt to achieve this objective.

## Overview of Cutaneous T-Cell Lymphomas (CTCLs)

Non-Hodgkin lymphomas can occur at extranodal sites in approximately 27% of cases, with the gastrointestinal tract and skin being the first and second most common sites of extranodal involvement (1). Most nodal non-Hodgkin lymphomas are B-cell derived, which is in contrast to the approximately 75–85% of primary cutaneous lymphomas that are T-cell derived (2–6). The incidence of CTCLs has been increasing (7); consequently, 4–8 people per million currently suffer from these cancers (8, 9). CTCL represents a series of skin-based neoplasms of T-cell origin, predominantly comprised of peripheral CD4^+^ T-cells. There are 12 distinct CTCL subtypes (Table 1), with mycosis fungoides (MF) being the most common (10). Primary cutaneous CD30^+^ T-cell lymphoproliferative disorders are the second most common, except in some countries in the Pacific where adult T-cell leukemia/lymphoma (ATL) ranks second (6, 11).

**Table 1:** List of primary CTCLs.

Study group	Frequency, %			Disease-specific 5-year survival^2^, %
	DACLG^2^	SEER16^5^	JSCS^6^	
Mycosis fungoides	61.5	54.1	51.7	88
Sézary syndrome	3.5	1.2	2.3	24
Primary cutaneous CD30^+^ T-cell lymphoproliferative disorders	26.0	14.4	14.3	
Lymphomatoid papulosis	16.1		4.5	100
Primary cutaneous anaplastic large-cell lymphoma	9.9		9.4	95
Adult T-cell leukemia/lymphoma^a^		0.1	20.0	
Subcutaneous panniculitis-like T-cell lymphoma	1.2	0.8	2.3	82
Primary cutaneous gamma/delta T-cell lymphoma	0.9		0.3	NR
Primary cutaneous CD4^+^ small/medium T-cell lymphoproliferative disorder^b^	2.7		1.7	75
Hydroa vacciniforme-like lymphoproliferative disorder^b^				
Primary cutaneous acral CD8^+^ T-cell lymphoma^b^				
Primary cutaneous CD8^+^ aggressive epidermotropic cytotoxic T-cell lymphoma	1.0		0.4	18
Peripheral T-cell lymphoma, NOS^a^	3.2	29.4	6.9	16
Total no. of CTCL	1,469	2,750	1,451	

*NR, not reached; CTCL, cutaneous T-cell lymphoma*.

*^a^A portion of these diseases is considered as primary CTCL*.

*^b^Provisional entity in World Health Organization classification (2016)*.

## MF/Sézary Syndrome (SS)

**Figure 1:**
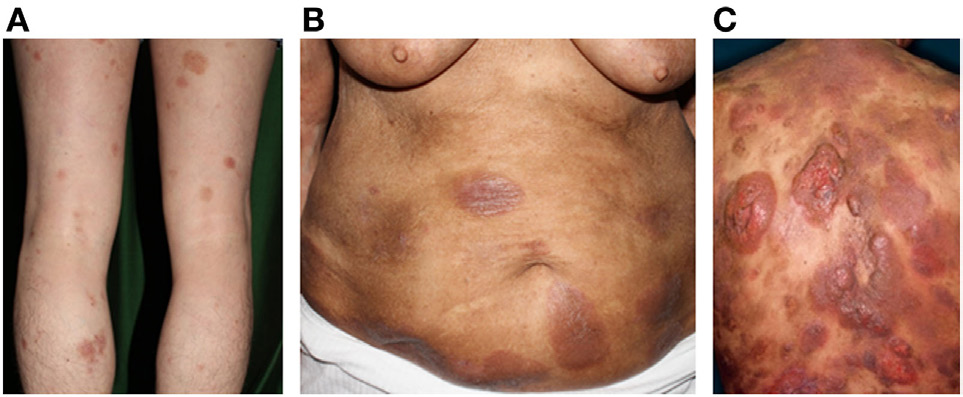
Clinical findings of mycosis fungoides/Sézary syndrome. **(A)** Patches, **(B)** plaques, **(C)** and nodules on the plaque. Written informed consent was obtained from each patient.

Mycosis fungoides and SS constitute the most common types of primary CTCLs. MF is characterized by erythematous patches, plaques, or tumors on the skin (Figure 1), with the involvement of lymph nodes, blood, and viscera also possible. MF can mimic benign inflammatory skin disorders, such as atopic dermatitis or psoriasis; thus, it is not unusual for MF to remain undiagnosed for years. Although MF is typically an indolent disorder, the disease may progress toward or exhibit *de novo* more advanced forms including tumors and erythroderma (>80% of the body surface area showing patches/plaques without overt leukemia). This can lead to lymph node or organ involvement, accompanied by increased morbidity and mortality. Patients are classified as having either early-stage (patches/plaques) or advanced-stage (tumors, erythroderma, lymph node, and/or visceral involvement) (12, 13). SS is the leukemic form of the disease, in which erythroderma is accompanied by measurable levels of malignant lymphocytes with cerebriform nuclei [i.e., Sézary cells (SC)] in the blood. Typical SC counts would be ≥1,000/μL, with a CD4/CD8 ratio of ≥10 and a loss of one or more T-cell antigens (CD4^+^CD7^−^ > 30% or CD4^+^CD26^−^ > 40%). Furthermore, CD30 expression is associated with a significantly reduced disease-specific survival and is often associated with histologically detectable large cell transformation, hallmarking a more aggressive clinical course (14).

In the past, SS has been considered a leukemic and aggressive variant of MF. However, a recent study determined that MF and SS arose from distinct T-cell subsets: SS from central memory T-cells and MF from skin-resident effector memory T-cells (15). CD158k/killer cell immunoglobulin-like receptor 3DL2 represents a specific marker for the evaluation of SC (16); in particular, CD4^+^ CD158k^+^ lymphocytes in blood from patients with SS correspond to the malignant clonal cell population (17). In addition, immunohistological finding of CD158k in affected skin is reported to distinguish SS from MF (18). Clonal malignant T-cells from the blood of patients with SS coexpress the lymph node homing molecules C–C motif chemokine receptor 7 (CCR7)/CD197 and CD62L/l-selectin, as well as the CD27 differentiation marker, a characteristic of central memory T-cells. This is consistent with the clinical presentation of peripheral blood disease, lymphadenopathy, and diffuse erythroderma of the skin. In contrast, T-cells from MF skin lesions do not express CCR7, l-selectin, and CD27, but strongly express CCR4 and cutaneous lymphocyte antigen (CLA)/CD162, characteristics of skin-resident effector memory T-cells. This difference in the putative origins between SS (central memory T-cell-derived) and MF (tissue-resident memory-derived) can explain their distinct clinical behaviors; central memory T-cells are long-lived, apoptosis-resistant cells that can be found in the peripheral blood, lymph nodes, and skin, whereas skin-resident memory T-cells remain in the skin and do not enter the general circulation. That MF and SS are derived from different T-cell precursors is also supported by comparative genomic hybridization and gene-expression profiling, demonstrating that the CTCL genotypes are distinct (19, 20). Overall, MF is characterized by gains on chromosomes 1 and 7 and losses on chromosome 9, whereas SS is characterized by gains on chromosomes 8 and 17 and losses on chromosome 10. A multiplatform genomic analysis of patients with SS detected (1) activating *CCR4* and caspase recruitment domain-containing protein 11 (*CARD11*) mutations in nearly one-third of patients; (2) deletion of zinc finger E-box binding homeobox 1 (*ZEB1*), encoding a transcriptional repressor essential for T-cell differentiation, in over one-half of patients; and (3) overexpression of interleukin 32 (IL-32) and interleukin-2 receptor subunit gamma in nearly all patients (21).

## Roles of Chemokines in Development of Skin Pathology

Malignant T-cells are suggested exhibit phenotypes of mature CD4^+^ memory T-cells, along with type 2 or 17 (Th2 or Th17 (22)) T-helper cell phenotypes, or be comprised of FOXP3 regulatory T-cells (Treg) (23, 24). Many chemokines are also reportedly expressed in the affected skin of patients with CTCL, suggesting that chemokine–receptor interactions play important roles in disease progression (25). Chemokine receptor expression on tumor cells in MF varies with disease stage. In the patch and plaque stages of MF, most infiltrating cells express CXC chemokine receptor (CXCR) 3/CD183 in the affected skin (26). CXCR3 binds three distinct ligands, namely CXC chemokine ligand (CXCL) 9/monokine induced by gamma interferon (MIG), interferon-gamma-inducible protein-10 (CXCL10/IP-10), and interferon-inducible T cell alpha chemoattractant (CXCL11/ITAC)/IP-9; all are expressed in the affected skin in the patch and plaque stages of MF (27–29). Various cell types express these chemokines including keratinocytes, dermal fibroblasts, and Langerhans cells. In the early stages of MF, expression of CXCL9, CXCL10, and CXCR3 is believed to be important for recruitment and accumulation of tumor cells in the skin (25, 28). However, later in the tumor stage, the tumor cells increase in size and tend to express CCR4 instead of CXCR3 (30). The expression levels of CXCL9 and CXCL10 also tend to be lower in the affected skin of patients with MF during the tumor stage than during the patch and plaque stages (31). Moreover, CCR6/CD196 and its ligand CCL20/macrophage inflammatory protein (MIP)-3α are upregulated in advanced CTCL (32). Tumor MF cells exhibit high levels of CCR7 (33), which is considered to be associated with loss of epidermotropism and migration to peripheral lymph nodes, which constitutively synthesize the CCR7 ligands, CCL19 and 21 (34). CCR7 is also expressed at high levels in SC (35), as mentioned in Section “MF/Sézary syndrome (SS)”.

Circulating SC and skin-infiltrating cells in SS also express CCR4 (30, 35, 36). CCR4-expressing T-cells were found in CTCL lesions along with high expression of two CCR4 ligands, namely CC chemokine ligand (CCL) 17/thymus and activation-regulated chemokine and CCL22/macrophage-derived chemokine (30). CCL17 is expressed by endothelial cells and keratinocytes in the affected skin of patients with MF and SS (30, 37). During the tumor stage of MF, serum CCL17 levels are much higher than those during the patch/plaque stages (37), suggesting the importance of CCL17–CCR4 interactions in tumor cell trafficking to the skin of these patients. CCL22 is expressed by dendritic-like cells and keratinocytes (30, 37). Serum CCL22 levels are significantly higher in patients with MF than in healthy controls or patients with psoriasis vulgaris (37).

CC chemokine ligand 27/cutaneous T cell attracting chemokine is a CCR10 ligand that is constitutively produced by activated keratinocytes in various diseases (38). CCR10 is expressed on the tumor cells of MF and SS (35, 39). Strong immunostaining of CCL27 has been observed in the affected skin of patients with MF compared to that of unaffected individuals (39, 40). Serum CCL27 levels and the number of circulating CCR10^+^ CD4^+^ cells are both increased in patients with MF compared to that of control patients (39). Therefore, CCR10–CCL27 interactions may also contribute to the migration of lymphoma cells to the affected skin in MF and SS. In addition to CCR4 and CCR10, expression of the receptor for CXCL12/stromal cell-derived factor 1, CXCR4, is observed in SC (36). CXCL12 is a chemoattractant for CXCR4-positive cells and is strongly expressed in the affected skin of patients with MF (41) and SS (36). Therefore, CXCL12–CXCR4 interactions may also facilitate the recruitment of lymphoma cells to the skin.

## Th2-Dominant Microenvironment

As the microenvironment in early-stage MF consists of non-malignant Th1 cells and CD8^+^ tumor-infiltrating T cells, MF and SS are considered Th2-type diseases, which are frequently accompanied by eosinophilia and high serum levels of IgE. In the early 1990s, peripheral blood mononuclear cells in patients with SS and non-leukemic CTCL were reported to be Th2 dominant (42, 43). In 1994, mRNA for Th2 cytokine was detected in the skin of patients with MF (44). T-cell clones from patients with SS were identified thereafter to have Th2-like properties (45). However, in the early stages of MF, affected skin and peripheral blood T-cells express a profile of Th1 cytokines (46, 47). The Th2 phenotype appears to be caused by leukemic T-cells, as culturing benign T-cells away from malignant clones reduces Th2 and enhances Th1 responses (48). The Th2-dominant microenvironment is advantageous for tumor cells, because interferon (IFN)-γ-producing Th1 cells enhance immune responses against the tumor. Indeed, IFN-γ has been shown to be effective for CTCL treatment (49, 50). Adenoviral-mediated gene therapies that increase expression of IFN-γ have also been used successfully in CTCL (51–53). CTCL cells can inhibit T-cell proliferation and suppress dendritic cell (DC) maturation by secretion of Th2 cytokines (54). Skin and nasal colonization with *Staphylococcus aureus* is common in patients with MF/SS; in particular, a Th2-dominant microenvironment may underlie this susceptibility to infection (55). Infections of *S. aureus* and sepsis also frequently occur in patients with CTCL (56). Accordingly, the major cause of death in patients with erythrodermic MF and SS is intravenous line sepsis, with *S. aureus* often being the causative microorganism (57).

In early-stage MF, signal transducers and activators of transcription (STAT) 4, the activation of which is required for Th1 differentiation, are overexpressed by IL-12 signaling *via* JAK2/TYK2 (58). In later stages, IL-2 and IL-15 signaling *via* JAK1 and JAK3 kinases activates STAT5, which increases the expression of oncogenic miR-155 (59) and subsequently inhibits STAT4 expression (60), resulting in a switch from Th1 to Th2 phenotype in malignant T cells. Downregulation of STAT4 is also induced by deficiencies in IL-12 expression (58, 61) and lack of the IL-12R β2 chain (58). During this switch, the expression of STAT6 is often upregulated in CTCL (60). STAT5 activation is seen in both early and late stages. Specifically, in the late stage, constitutive STAT5 activation is induced by cytokine-independent JAK1/JAK3 signaling (59). In the advanced stage, such constitutive STAT3 activation, which increases survival and resistance to apoptosis and promotes Th2 and Th17 phenotypes, is induced by an IL-21 autocrine signaling loop (62), the presence of IL-7 and IL-15 in the microenvironment (63), and/or constitutive cytokine-independent activation of JAK1 and JAK3 signaling (64, 65). Moreover, GATA3, a transcriptional regulator of Th2-cells, is overexpressed in SC *via* proteasome dysregulation (66).

## Cancer-Associated Fibroblasts

Fibroblasts are crucial components of the tumor microenvironment, promoting the growth and invasion of cancer cells through various mechanisms (67). The fibroblasts in the affected skin of patients with advanced CTCL promote a Th2-dominant microenvironment by augmenting Th2 and attenuating Th1 immune responses. Increased expression of CCL26/eotaxin-3 is observed in the dermal fibroblasts, keratinocytes, and endothelial cells of the affected skin of patients with advanced MF (68). In addition, serum CCL26 and CCL11/eotaxin-1 levels were shown to be higher in patients with CTCL than in healthy control patients, which correlated with serum soluble interleukin-2 receptor (sIL-2R) levels. However, CCR3/CD193, a receptor for CCL26 and other ligands, is not expressed on lymphoma cells in MF or SS (69). Because mRNA for CCR3 is detected in affected skin (68) and CCR3 is expressed on eosinophils and subpopulations of Th2 cells (70, 71), CCL26 and CCL11 are believed to support the Th2-dominant microenvironment in MF and SS disease lesions (25).

Periostin constitutes an extracellular matrix protein that is expressed in several cancers (72); it is prominent in the stromal area during the patch and plaque stages of MF, but decreases during the tumor stage (73). Fibroblasts are reportedly the source of periostin in MF (74). IL-4 and IL-13 can induce periostin secretion by dermal fibroblasts, periostin mediates thymic stromal protein (TSLP) production by keratinocytes, and TSLP subsequently activates immature myeloid DCs, which modulate Th2 immune responses *via* CCL17 production (75). Serum (76) and plasma (77) TSLP levels are increased in patients with CTCL, suggesting that TSLP contributes to the Th2-dominant microenvironment in MF lesions. TSLP also induces the growth of CTCL cells (74). Therefore, periostin can directly stimulate the growth of CTCL tumor cells in addition to inducing a Th2-dominant environment in CTCL tumors.

Expression of herpesvirus entry mediator (HVEM)/CD270, a member of the tumor necrosis factor-receptor superfamily, on dermal fibroblasts in the affected skin of patients with MF and SS is decreased as the disease progresses. In addition, low HVEM expression on dermal fibroblasts in the affected skin of patients with advanced CTCL attenuates the expression of Th1 chemokines, resulting in Th2-dominant microenvironments. This occurs because the interaction between HVEM and tumor necrosis factor superfamily member 14 (also termed LIGHT)/CD258 on dermal fibroblasts increases the secretion of CXCL9–11, which are chemokines that recruit CXCR3-positive Th1 cells (29).

## Tumor-Associated Macrophages (TAMs)

Macrophages constitute a major component of the leukocyte infiltrate in the tumor microenvironment (78), in which they are termed TAMs. TAMs usually comprise polarized M2 macrophages that contribute to an immune-suppressive environment and promote tumor cell growth (79). CD163 is recognized as a marker for TAMs. As with many malignancies (80), the presence of M2 macrophages in the affected skin of patients with MF has been correlated with patient prognosis (81, 82), and the presence of M2 macrophages has been correlated with lymph node staging (83); this suggests that TAMs play a significant role in MF pathogenesis. Serum sCD163 levels in patients with CTCL are significantly higher than those in normal controls and they significantly correlate with serum sIL-2R levels. TAMs are believed to play a role in the formation of CTCL by secreting various chemokines (73, 82, 84). Periostin-stimulated macrophages produce CXCL5 and CXCL10 (73), which correlates with MF tumor formation in a xenograft CTCL mouse model (84). CCL18/alternative macrophage activation-associated CC chemokine 1/MIP-4 is secreted by M2 macrophages (85) and binds to its receptor (i.e., CCR8) on Th2 cells (86). The expression of CCR8 on MF or SS tumor cells has not been reported, although the mRNA expression of CCR8 is known to be upregulated in patients with SS (21). TAMs are known to express CCL18 in the skin of patients with CTCL (87, 88). Serum CCL18 levels were significantly higher in patients with CTCL than in healthy controls, and these levels significantly correlated with modified severity-weighted assessment scores, serum sIL-2R, lactate dehydrogenase, Th2 cytokines, and chemokines (88). Furthermore, high serum levels of CCL18 were associated with poor patient prognosis (88). In the affected skin of patients with MF/SS, TAMs highly express CD30, which is the target of the anti-CD30 antibody–drug conjugate, brentuximab vedotin (89).

## Dendritic Cells

Dendritic cells are antigen-presenting cells with a unique capacity to induce primary immune responses (90). By secreting Th2 cytokines, CTCL cells can suppress the maturation of DCs (54). Notably, IL-10 downregulates DC functions and may promote tolerance by skin DCs, rather than immune defense (91). Immature DCs can induce tolerance by presenting antigens to T-cells without appropriate costimulation. A significant increase in various DC subsets is seen in the affected dermis of patients with MF/SS, with the majority being immature CD209/DC-specific ICAM-3 grabbing non-integrin (DC-SIGN)-positive DCs. Increases in CD208/DC-lysosome-associated membrane glycoprotein-positive DCs (i.e., mature DCs) and CD303/blood dendritic cell antigen 2-positive DCs (i.e., plasmacytoid DCs) are also observed, but the numbers of cells expressing CD208 or CD303 are few, suggesting that many DCs in the dermis of CTCL lesions are immature. Increased number of immature DCs in CTCL lesions may be important for immunological tolerance against malignant T-cells (92). However, some CD208-positive, mature DCs may attempt to mount an immune response against the cancer cells, as mature CD208-positive DCs are elevated in the skin draining lymph nodes of patients with MF (83).

## Other Key Players

The keratinocytes in the affected skin of patients with MF/SS release multiple chemokines including CCL17, CCL26, CCL27, CXCL9, and CXCL10, which help to attract T-cells to the epidermis, as mentioned above. Nerve growth factor (NGF) expression is also elevated in the affected skin of patients with SS, which stimulates the sprouting of nerve fibers. NGF is associated with the severity of pruritus in atopic dermatitis (93), and serum NGF levels are elevated in patients with SS (94). The enhanced expression of NGF is supposedly associated with pruritus in SS.

Mast cells also serve as critical stimulators of the tumor microenvironment (95). Patients with CTCL have increased number of mast cells in their affected skin and this correlates with disease progression (96). Moreover, in a model of cutaneous lymphoma, tumor growth in mast cell-deficient mice was significantly decreased. Therefore, mast cells represent key players in the development of CTCL.

Th22 cells, which produce IL-22 but not IFN-γ, IL-4, or IL-17, express CCR6, CCR4, and CCR10, thus enhancing skin infiltration. IL-22 mediates host defenses against bacterial infection (97). The affected skin of patients with MF/SS expresses high levels of IL-22 and low levels of IL-17 (32). A case of SS reportedly also had high IL-22 expression that was modulated by systemic bacterial infections (98). The serum levels of IL-22 and IL-22-induced CCL20 are increased in patients with MF/SS and are associated with disease severity (32); this suggests an important role of IL-22 in establishing the tumor microenvironment in MF and SS.

Myeloid-derived suppressor cells (MDSCs) are also recognized as key players in tumor immune escape mechanisms (99). The progression from early patch/plaque lesions to tumors in MF is related to an increase in MDSCs (100). Therefore, MDSCs play a role in MF progression by decreasing antitumor immune responses.

T-cell exhaustion *via* immune checkpoints also constitutes an important factor underlying tumor survival. The expression levels of PD-1 (101, 102), PD-L1 (102), CTLA-4 (103), and ICOS (104) have been described at different stages of the disease, suggesting a role for immune checkpoint inhibitor therapies.

## Treatment

There is currently no cure for CTCL, thus treatment is aimed primarily at improving symptoms and quality of life and maintaining remission. Therapies are tailored to the individual patient, based on age, performance status, extent of disease burden, rate of disease progression, and prior treatments (105). A typical MF progression starts at the patch and plaque stage and then advances to dermal-based tumors over many years. Effective immune control in the initial disease stages can slow disease progression. Hughes et al. reported that chemotherapy shortens the median time until the next treatment in patients with MF/SS (106). Multiagent chemotherapy often induces immunosuppression, which leads to an increased risk of infection and poor tolerance (107). Therefore, chemotherapy should be limited until all other options are exhausted. In comparison, IFN and histone deacetylase inhibitors afford greater times to next treatment than those from chemotherapy.

Both IFN-α and IFN-γ represent effective clinical treatments for CTCLs, including MF, *via* their cytotoxic and immunological effects on tumor-associated T-cells (108–110). A meta-analysis suggested that the overall response rate (ORR) to IFN-α was 70% (109). In all stages of MF, IFN-α achieves a superior time to next treatment compared to that of chemotherapy (106). IFN-γ shifts the Th2-dominant tumor microenvironment to a Th1 environment, as mentioned above. IFN-α2a and IFN-γ have been shown to decrease the expression and production of CCL17 and CCL18 and increase those of CXCL10 and CXCL11. Furthermore, subcutaneous administration of IFN-α increased the number of CXCL11-producing cells in the affected skin of patients with advanced MF (111).

Toll-like receptor (TLR) agonists induce anticancer effects by stimulating the innate immune system. Imiquimod is a topical immunomodulator that stimulates Th1 responses by activating TLR7 on plasmacytoid DCs, which leads to the production of IFN-α, IL-12, and tumor necrosis factor-α (112). The effectiveness of topical imiquimod has been reported in early-stage (113–115), folliculotropic, and tumor-stage MF (116). Resiquimod, a TLR7/8 agonist, is also effective for early-stage MF (117). TLR8 is expressed by myeloid-derived DCs, which are the most abundant DCs in human skin. Resiquimod, but not imiquimod, potently activates these cells (118).

The acetylation of histones plays a critical role in gene expression regulation (119). Histone acetylation and deacetylation control gene transcription and are mediated by histone acetyltransferases and deacetylases, respectively. Histone deacetylase inhibitors enhance the acetylation of histones and non-histone proteins and can induce apoptosis (120). Histone deacetylase inhibitors are potential therapeutic agents for the treatment of lymphoid neoplasms (121–124). Pruritus relief has also been reported with these inhibitors (121, 122, 124–126), supposedly through the reduction in the levels of IL-31-expressing T-cells (127).

Brentuximab vedotin (mentioned above) is an antibody–drug conjugate, in which an anti-CD30 monoclonal antibody is linked with the anti-tubulin agent, monomethyl auristatin E (128). Brentuximab vedotin is effective in the treatment of CD30-positive relapsed/refractory Hodgkin’s lymphoma (129) and anaplastic large cell lymphoma (130). In a phase II study for MF/SS with variable CD30 expression levels, an ORR of 70% was observed with brentuximab vedotin (127). In addition, a significant improvement in objective response was observed in a randomized, phase III clinical trial (131).

Mogamulizumab, a defucosylated humanized anti-CCR4 antibody that was first approved for relapsed ATL, as described in further detail in Section “Adult T-cell leukemia/lymphoma,” is also effective for CTCL including MF/SS (132, 133), and approved for relapsed or refractory CCR4-positive CTCL. In addition, an anti-CD158k monoclonal antibody, IPH4102, has also recently been developed (134), for which clinical studies in CTCL are ongoing (135). Lenalidomide (136), bortezomib (137), and immune checkpoint blockade are also under investigation.

## Primary Cutaneous CD30^+^ T-Cell Lymphoproliferative Disorders

Primary cutaneous CD30^+^ T-cell lymphoproliferative disorders (PC CD30^+^ T-LPD) constitute the second most common form of CTCL, representing approximately 30% of all cutaneous lymphomas (2). They comprise a spectrum of diseases from lymphomatoid papulosis (LyP) to primary cutaneous anaplastic large-cell lymphoma (PCALCL) (138). The expression of CD30, a cytokine receptor belonging to the tumor necrosis factor receptor superfamily, by atypical T-cells is the common immunophenotype of this disorder.

**Figure 2:**
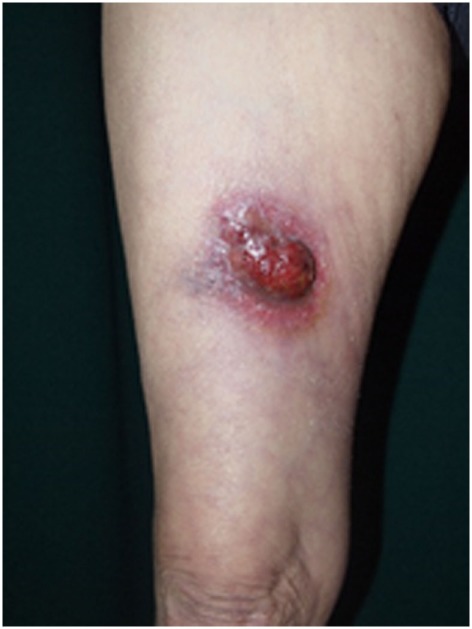
Clinical findings of primary cutaneous CD30^+^ T-cell lymphoma. Eroded tumor is seen on the right thigh. Written informed consent was obtained from the patient.

Primary cutaneous anaplastic large-cell lymphoma is characterized by large T-cells with prominent nuclear pleomorphisms along with CD30 expression by more than 75% of the tumor cells (2). A single tumor or a group of firm nodules is seen clinically (Figure 2). PCALCL was established as a distinct form of ALCL because its clinical course, phenotype, and genotype are significantly different from those of systemic ALCL, including ALK-positive and ALK-negative forms (139–141). Moreover, IFN regulatory factor-4 translocations are reported to be specific for PCALCL (142). In contrast to that of systemic ALCL, the prognosis of PCALCL is reportedly excellent (143), with the exception of cases in Japan that appear to have a less favorable prognosis (144). PCALCL arising on the legs tends to produce poorer outcomes (145). The typical histology of PCALCL is a circumscribed nodular infiltrate of cohesively arranged large lymphoid cells that extends into the deep dermis or hypodermis. Neutrophil-rich and eosinophil-rich variants have been noted and appear to be associated with immunodeficiency (146). The abundant infiltration of neutrophils can be explained by the release of IL-8, a potent neutrophil chemoattractant, from the tumor cells (147).

The tumor cells in PCALCL possess an activated T-cell phenotype and express CD2, CD4, and CD45RO, with a loss of CD2 and CD5 occurring variably. CD3 may be lacking or expressed at lower levels owing to genetic alterations in the T-cell receptor (TCR) coding regions on chromosome 1 in the tumor cells (148). Additionally, CD25/IL-2R, CD71, human leukocyte antigen–antigen D related, and CLA/CD162, as well as cytotoxic proteins, such as T-cell intracellular antigen 1 (TIA-1), granzyme B, and perforin, are expressed in half of PCALCL cases. PCALCL is often negative for epithelial membrane antigen, which differentiates it from systemic ALCL. Numerous quantities of TAMs are also present.

As opposed to MF/SS, the tumor cells of PCALCL express CCR3. CCL11, a CCR3 ligand, is also expressed by PCALCL cells and is detected in the connective tissue cells in the tumor. The CCR3^+^ tumor cells abundantly express IL-4 but not IFN-γ (69). The expression of both CCL11 and CCR3 on the tumor cells can lead to homotypic aggregation, which can be observed as cohesive clusters of tumor cells, a characteristic finding in ALCL (149). As CCR3 is also expressed on eosinophils and subpopulations of Th2 cells (70, 71), CCR3^+^ cells secreting CCL11 and IL-4 may produce a Th2-dominant microenvironment, which is suitable for tumor growth.

Lymphomatoid papulosis was first described by the dermatologist, Warren L. Macaulay, as a chronic recurrent, self-regressing papulonodular skin eruption with histologic features of a malignant lymphoma (138). Five histological variants (types A to E) are recognized as original variants in the updated World Health Organization classification of 2016 (10). LyP type A is the most common subtype, accounting for 75% of LyP cases (150). Type A is characterized by wedge-shaped dermal infiltrates with scattered large CD30^+^ cells. Histiocytes, eosinophils, and neutrophils comprise the background inflammatory cells. Type B shows epidermotropic infiltrates of small to medium-sized lymphocytes with variable CD30 expression and atypical chromatin-dense nuclei. Type C shows nodular cohesive infiltrates of large CD30^+^ pleomorphic or anaplastic lymphocytes. Type D shows epidermotropic infiltrates of atypical, small to medium-sized pleomorphic CD8^+^ cytotoxic cells (151). Type E shows angioinvasive infiltrates of mainly medium-sized pleomorphic CD30^+^ cells (152). Vascular occlusion by atypical lymphocytes and/or thrombi, hemorrhage, ulceration, and extensive necrosis are observed. LyP can persist for years or decades, but is not life-threatening (143, 153). However, some patients with LyP can develop secondary lymphoid neoplasms, in particular MF, Hodgkin’s lymphoma, and cutaneous or nodal CD30^+^ ALCL (140, 146, 154). Surgical excision or radiation therapy is the recommended therapy for solitary or grouped lesion(s) of PCALCL, whereas methotrexate is the most prescribed therapy for multifocal lesions (138). The brentuximab vedotin (128) has been granted breakthrough therapy designation; in addition, bexarotene, a retinoid X receptor-specific agonist, has also been shown to be effective for both PCALCL (ORR: 50%) and LyP (ORR: 60%) in clinical trials (155). HDAC inhibitors (156), crizotinib, an ALK inhibitor (157), and anti-PD-1 are under investigation.

## Adult T-Cell Leukemia/Lymphoma

**Figure 3:**
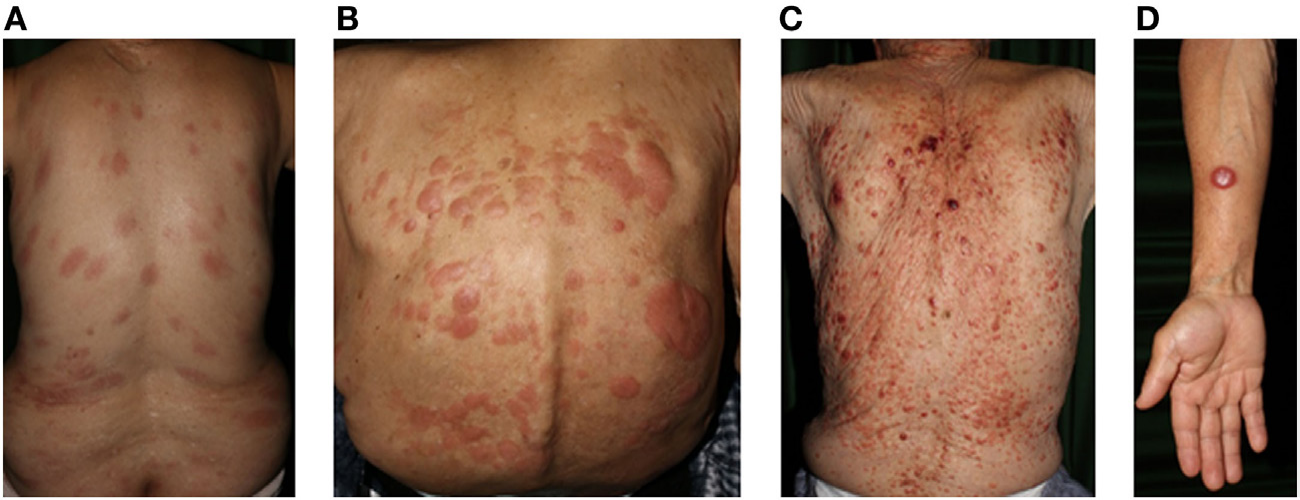
Clinical findings of adult T-cell lymphoma/leukemia. **(A)** Patch, **(B)** plaque, **(C)** multiple nodulotumoral lesion, and **(D)** tumor. Written informed consent was obtained from each patient.

Adult T-cell leukemia/lymphoma is a distinct T-cell malignancy caused by human T-lymphotropic virus type I (HTLV-1). HTLV-I infections are endemic in many parts of the world including southwest Japan, the Caribbean basin, and parts of central Africa and South America. Neoplastic T-cells are usually CD4^+^CD25^+^CCR4^+^ (158). The general characteristics of ATL are lymphadenopathy, hepatosplenomegaly, hypercalcemia, abnormal peripheral blood lymphocytes with multilobulated nuclei, and skin lesions (Figure 3).

There are four clinical subtypes of ATL (159): acute, lymphoma, chronic, and smoldering, based on peripheral blood involvement, organ complications, and laboratory examinations. Patients with ATL can be stratified into two groups: aggressive, which consists of the acute, lymphoma, and unfavorable chronic types, and indolent, which consists of the favorable chronic and smoldering types. The chronic type is separated into the favorable and unfavorable subgroups according to significant prognostic factors. This stratification is important for treatment selection, with most patients with aggressive ATL being given systemic chemotherapy, whereas those with indolent ATL are given topical therapy or are placed on observation.

Cutaneous involvement is frequently observed in patients with ATL at 30–70% (160, 161), regardless of ATL subtype. Cutaneous manifestation in the smoldering type of ATL has been suggested to reflect poor prognosis (162), and cutaneous ATL was recently proposed to include the lymphoma type as an extranodal variant (163). The majority of skin lesions are caused by the direct invasion of ATL tumor cells, forming various types of eruptions (164). In addition to these primary invasive lesions, patients with ATL may present with secondary inflammatory or infectious lesions (165). Compared to those of peripheral blood tumor cells, skin-infiltrating ATL tumor cells exhibit enhanced characteristics, such as increased expression of chemokine receptors. The interaction between chemokines and chemokine receptors drives T-cell migration and activation, which plays a critical role in the pathogenesis of various neoplastic and inflammatory disorders. ATL cells produce several chemokines including CCL3/MIP-1α, CCL4/MIP-1β (166), CCL2/monocyte chemoattractant protein-1 (MCP-1) (167), and CCL1/I-309 (168), as well as several chemokine receptors, including CCR4 (158, 169), CCR7 (170), and CCR8/CDw198 (168). Overexpression of chemokine CCL1 and its receptor, CCR8, contributes to autocrine anti-apoptotic effects ATL cells (168). Increased CCR7 expression is associated with lymphoid organ infiltration (170).

Adult T-cell leukemia/lymphoma cells not only express CCR4 but also its ligands, CCL17 and CCL22 (171). Neoplastic T-cells that highly express the Th2 chemokine receptor, CCR4, are found in the peripheral blood and affected skin of patients with ATL. In CTCL, extravasation of lymphoma cells into the skin is mediated by CCL17 and CCL22 released from epidermal cells (30). In contrast, one of the major sources of CCL17 in the affected skin of patients with ATL is the tumor cell itself (171). Moreover, CCL17 and CCL22 can also attract CCR4-expressing Treg cells, which may further suppress cytotoxic T-cells and prevent tumor immunosurveillance of the ATL cells (165). As ATL cells share the CD4^+^CD25^+^CCR4^+^ phenotype with Treg cells, ATL cells have been postulated as being Treg cells. In addition to CD25 and CCR4, ATL cells express CTLA-4 and FoxP3, both of which are expressed in Treg cells (172, 173). However, whether ATL cells can function as Treg cells is controversial because tumor cells possess very limited regulatory ability (174).

Th17 cells play an important role in cutaneous innate immunity. Th17-derived cytokines stimulate keratinocytes to produce antimicrobial peptides (175). ATL tumor cells can reduce the number and/or function of Th17 cells. Studies have shown that cellular immune responses are greatly impaired in patients with ATL, and ATL cells have been shown to secrete immunosuppressive cytokines such as IL-10 and transforming growth factor-β1 *in vitro*. In particular, ATL cells, as well as Treg or Th2 cells residing in the blood, produce IL-10, thereby suppressing Th17 activity (176). IL-17 enhances the synthesis of various antimicrobial peptides, such as human β-defensin 2, LL-37 (177), and S100A7, in keratinocytes. These peptides are active against fungi, such as those causing ringworm (178). More than 60% of patients with ATL have tinea pedis/unguium/corporis, candidiasis, or other cutaneous fungal infections (165). Other skin infections may occur in these patients in addition to superficial fungal infections. It has been reported that scabies is sometimes superimposed on the skin lesions of patients with ATL (179).

Programmed cell death (PD)-1/CD279 constitutes a cell surface receptor that suppresses the immune system. PD-1 expression on HTLV-1-specific cytotoxic T-cells is dramatically upregulated in HTLV-1 carriers and patients with ATL (180). PD-1 is expressed at high levels on CD4^+^ neoplastic and non-neoplastic cells, but not on CD8^+^ cells (181). Because normal CD4^+^ T-cells can be infected with HTLV-1, they can sometimes express PD-1, leading to immunosuppression. Moreover, it is noteworthy that PD-L1 is expressed in ATL cells (181). Expression of both PD-1 and PD-L1 by the ATL cells suggests a self-destructive state of the tumor cells. However, it may be more important that the PD-L1 expressed by the tumor cells suppresses the function of PD-1-expressing normal CD4^+^ T-cells, resulting in immune evasion. Of note, 25% of patients with ATL have structural variations in the 3’-region of the gene for PD-L1, which leads to marked elevations of aberrant *PDL1* transcripts (182).

The fact that the tumor cells express CCR4 provides a therapeutic strategy for ATL. The anti-CCR4 monoclonal antibody mogamulizumab markedly enhances antibody-dependent cellular cytotoxicity and has been approved for the treatment of patients with CCR4-positive ATL, peripheral T-cell lymphoma, and CTCL. In a phase II trial of patients with relapsed CCR4-positive ATL, the ORR was 50%, with a complete response rate of 30% (183). Mogamulizumab is more effective against the peripheral blood tumor cells than those in the skin and lymph nodes. Cutaneous adverse reactions (CARs) are frequently observed during treatment (183, 184) and are supposedly indicative of favorable prognoses in ATL (185); a reduction in Treg by mogamulizumab is believed to induce CARs (186, 187). Recently, pretransplantation mogamulizumab has been reported to increase the risk of severe acute graft-versus-host disease (188, 189), and non-relapse mortality is significantly higher in patients with pretransplantation mogamulizumab. Therefore, mogamulizumab should be carefully considered and monitored for patients with ATL who are eligible for allogeneic hematopoietic stem-cell transplantation.

## Panniculitis-Like T-Cell Lymphoma

Subcutaneous panniculitis-like T-cell lymphoma (SPTCL) with α/β phenotype and SPTCL with γ/δ phenotype have been recognized as unique entities, considering their clinical, histological, and immunological characteristics (2, 190, 191). The term SPTCL is now used exclusively for cases with the α/β T-cell phenotype, whereas those of the γ/δ T-cell phenotype have been reclassified as primary cutaneous gamma/delta T-cell lymphoma (PCGD-TCL) (2). The differential diagnosis of these two diseases is important, as each has a different prognosis and therapeutic strategy. In addition, both entities should be differentiated from other types of malignant lymphoma with preferential subcutaneous involvement and from other forms of lobular panniculitis, especially lupus panniculitis (192, 193).

## Subcutaneous Panniculitis-Like T-Cell Lymphoma

Patients with SPTCL present clinically with multiple nodules or deeply seated plaques without ulceration. The skin lesions usually involve the legs, arms, and trunk. Systemic symptoms, such as pyrexia, fatigue, and weight loss, and laboratory abnormalities, including cytopenia and elevated liver function tests, are commonly observed. Hemophagocytic syndrome (HPS) is observed in <20% of patients (194). Dissemination to extracutaneous sites rarely occurs. As many as 20% of patients have associated autoimmune disease, which is commonly systemic lupus erythematosus (194).

The histopathological findings in SPTCL are dense, nodular, or diffuse subcutaneous infiltrates with a pattern similar to lobular panniculitis. The epidermis is not typically involved. The rimming of individual fat cells by neoplastic T-cells is a curious finding, although it is not diagnostic (193). The neoplastic T-cells are interspersed with small reactive lymphocytes and many histiocytes, whereas other inflammatory cells, including neutrophils and eosinophils, as well as the plasma cells and plasmacytoid DCs that are common in lupus panniculitis (195, 196), are usually lacking (193). High-throughput sequencing of the TCR genes can assist in the diagnosis of SPTCL (192). The neoplastic cells have a mature CD3^+^CD4^−^CD8^+^ T-cell phenotype and express cytotoxic proteins, such as granzyme B, TIA-1, and perforin (194). Although the exact mechanisms that neoplastic cells utilize to migrate into the hypodermis are still mostly unknown, CCR5 expression on neoplastic cells and its ligands, CCL3, CCL4, and CCL5, which can be secreted from immunologically activated adipocytes, may contribute to the pathogenesis of SPTCL (197, 198).

The differential diagnosis of SPTCL includes both PCGD-TCL and lupus panniculitis. Differentiation is critical because PCGD-TCL with panniculitis-like features generally has a poor prognosis and requires systemic chemotherapy. In contrast, SPTCL has an excellent prognosis, especially in the cases without HPS (194). Both SPTCL and PCGD-TCL have nodular skin lesions with panniculitis-like features and rimming of fat cells. In contrast to that of SPTCL, PCGD-TCL involves ulceration of the hypodermis, dermis, and/or epidermis (194). Expression of βF1, but not TCRγ/δ or CD56, is useful to differentiate between SPTCL and PCGD-TCL.

Multiagent chemotherapy is not recommended as a first-line treatment for SPTCL without HPS. Systemic corticosteroids or other immunosuppressive agents, such as cyclosporine or methotrexate, are preferred, which is also the case with relapsing disease (199–201). Oral bexarotene has also shown good response rates (202).

## Primary Cutaneous Gamma/Delta T-Cell Lymphoma

Primary cutaneous gamma/delta T-cell lymphoma is a lymphoma composed of a clonal proliferation of mature, activated γ/δ T-cells with a cytotoxic phenotype. Most patients present with deep dermal or subcutaneous plaques or tumors, either with or without epidermal ulceration and necrosis (194, 203, 204). The skin lesions are often generalized and involve the extremities. Some patients may present with a single tumor, or scaly patches/plaques, clinically resembling early-stage MF (204). The involvement of mucosal and other extranodal sites is frequently noted, although lymph nodes, spleen, and bone marrow are rarely involved (204, 205). Most patients present with systemic symptoms including B symptoms. PCGD-TCL is frequently accompanied by HPS, particularly in patients with panniculitis-like tumors (194, 203). Chronic antigenic stimulation has been hypothesized to be involved in the pathogenesis of PCGD-TCL (206). PCGD-TCL is also associated with opportunistic infections in patients with congenital or acquired immunosuppression and autoimmunity (207–209).

The lymphoid infiltrates have a variable histological pattern and may be epidermotropic, dermal, and/or subcutaneous (203, 204). In contrast to that of SPTCL, a pure panniculitic pattern is rarely observed (204), and variable patterns can be found in skin biopsies obtained from different sites or different parts of the same biopsy (190, 203, 204). Lichenoid or vascular interface dermatitis-like patterns of epidermal infiltration may occur, which may be associated with intraepidermal vesiculation and necrosis (204). Panniculitis-like lesions may show the rimming of fat cells observed in SPTCL. Angiocentricity, angiodestruction, and tissue necrosis may be seen. Hemophagocytosis may be present, especially in cases with HPS. The tumor cells have a characteristic phenotype of TCR γ/δ^+^, βF1^−^, CD3^+^, CD2^+^, CD5^−^, and CD56^+^, with a strong expression of cytotoxic proteins. PCGD-TCL with subcutaneous panniculitis-like infiltrate preferentially derives from the V2 subtype (205). PCGD-TCL is resistant to multiagent chemotherapy. The effectiveness of hematopoietic stem cell transplantation has been reported in some patients with PCGD-TCL (204, 210, 211).

## Primary Cutaneous CD4^+^ Small/Medium T-Cell Lymphoproliferative Disorder

Primary cutaneous small/medium-sized T-cell lymphoma (PCSM-TCL) has recently been reclassified as primary cutaneous small/medium-sized T-cell lymphoproliferative disorder (PCSM-TCLPD) because of its indolent behavior and uncertain malignancy (10). PCSM-TCL was originally associated with a favorable 5-year survival rate of 60–80% (2). However, fatal outcomes have not been documented in subsequent reports (212, 213).

Primary cutaneous small/medium-sized T-cell lymphoproliferative disorder characteristically presents with a single lesion on the head, neck, or upper arms, but rarely presents as multiple papules, plaques, or tumors (212, 214). Histopathologically, PCSM-TCLPD is characterized by many small- to medium-sized CD3^+^CD4^+^CD8^−^ T-cells, with a small number of large CD4^+^ pleomorphic T-cells and variable admixtures of CD8^+^ T-cells, B-cells, histiocytes, plasma cells, and eosinophils (2).

The few, large pleomorphic CD4^+^ T-cells in PCSM-TCLPD express PD-1, BCL6, and CXCL13 (215), all of which are expressed on a particular germinal center T-cell subset, termed follicular helper T (TFH) cells. TFH cells are important in germinal center formation and plasma cell development. The expression of PD-1, BCL6, and CXCL13 by these large CD4^+^ T-cells suggests that PCSM-TCLPD originates from TFH cells (215). PD-1 is typically expressed by atypical cells in PCSM-TCL and pseudo-T-cell lymphomas (216). The clinical presentation, pathological features, and immunohistochemical findings of PCSM-TCLPD are very similar to those of pseudo-T-cell lymphomas (217, 218). The demonstration of a T-cell clone and loss of pan-T-cell antigens are useful diagnostic criteria for PCSM-TCL (218). The staining pattern for nuclear factor of activated T-cells, cytoplasmic 1 is also reported to be useful for the differential diagnosis between PCSM-TCLPD and pseudo-T-cell lymphomas (219), where NFAT1c nuclear staining indicates PCSM-TCLPD and cytoplasmic staining indicates pseudo-T-cell lymphoma. The cytoplasmic staining pattern is also seen in MF, ALCL, and LyP. The clinical behavior of PCSM-TCLPD is almost always indolent, with most patients showing localized disease. Treatment with local therapies, such as excision or radiation therapy, is often curative (214, 220, 221).

## Hydroa Vacciniforme-Like Lymphoproliferative Disorder (HVLL)

Typical hydroa vacciniforme (HV) is characterized by light-induced herpetiform vesiculopapules on the sun-exposed areas. The eruptions form crusts and then heal to leave varicelliform scars. Systemic symptoms are absent, and the disease usually improves spontaneously in adolescence and young adulthood (222). Routine laboratory tests are normal. Since the first report in 1986 (223), peculiar HV-like eruptions have been recognized in children mainly from Asia and Central and South America. HVLL was included for the first time in the 2008 World Health Organization classification of tumors of hematopoietic and lymphoid tissues (224). HVLL is defined as an Epstein–Barr virus (EBV)-positive CTCL that occurs in children and less often in young adults (225). Unlike typical HV, HVLL eruptions become more severe with age, presenting with marked facial edema and vesiclopapules followed by ulceration and crusting. Systemic symptoms, including high-grade fever and liver damage, are usually present. Hepatosplenomegaly and lymphadenopathy are frequently observed during the acute phase. The lesions are associated with EBV infection and frequently possess monoclonal rearrangements of the TCR genes (226, 227). Although the skin lesions are not limited to sun-exposed areas, there is an increased occurrence during the summer. Most cases have a CD8^+^ T-cell phenotype (228), whereas a small number of cases have been reported to have a natural killer-cell phenotype (229, 230). Regardless of cell-type derivation, the lymphoid cells are positive for cytotoxic markers, such as granzyme B and TIA-1 (231).

## Severe Mosquito Bite Allergy

An associated cutaneous disorder is a severe allergy/hypersensitivity to mosquito bites (232). It is defined as an EBV^+^ NK-cell lymphoproliferation that is characterized by high fever, ulcers, skin necrosis, and deep scarring, with the potential to progress into overt NK/T-cell lymphoma or aggressive NK-cell leukemia in the protracted clinical course (233). Severe mosquito bite allergy was included for the first time in the 2017 World Health Organization classification of tumors of hematopoietic and lymphoid tissues (234).

## Primary Cutaneous Acral CD8^+^ T-Cell Lymphoma

Primary cutaneous acral CD8^+^ T-cell lymphoma is characterized as a solitary, slow-growing nodule without prior patches or plaques (235), but with precedence of bilateral, symmetrical disease and recurrent disease (236). Most cases appear on the ear, although other peripheral locations, such as the nose, hands, and feet, have been noted (237).

Primary cutaneous acral CD8^+^ T-cell lymphoma and PCSM-TCLPD are often indistinguishable morphologically. Moreover, the overt clinical features of both diseases are similar, such as targeting adults, a preference for the face and neck, solitary tumors without ulceration, and an indolent behavior. However, T follicular markers, such as CD10, Bcl-6, PD-1, and CXCL13, which are expressed on neoplastic cells of PCSM-TCLPD, are negative in primary cutaneous acral CD8^+^ T-cell lymphoma (236). Granzyme B expression is also typically negative in the latter (238). The clinical course for primary cutaneous acral CD8^+^ T-cell lymphoma is invariably indolent; cutaneous relapse may occur, but there have been no reports of progression to extracutaneous sites, and overtreatment should be avoided (238). Localized therapy, such as topical steroids, radiotherapy, and surgical excision, or careful monitoring, is preferred. IFN, psoralen-ultraviolet A phototherapy, and methotrexate have been used for patients with multifocal cutaneous disease (238).

## Primary Cutaneous CD8^+^ Aggressive Epidermotropic Cytotoxic T-Cell Lymphoma

Primary cutaneous CD8^+^ aggressive epidermotropic cytotoxic T-cell lymphoma (PCAETCL) is characterized by disseminated, rapidly developing papules, plaques, and nodules with central ulceration or necrosis. PCAETCL may spread to other visceral organs including the lungs, testes, central nervous system, and oral mucosa (239–241); it carries an overall poor prognosis. However, the lymph nodes are rarely involved. Histological findings demonstrate prominent epidermotropism, with necrotic keratinocytes and ulceration (240). Dermal infiltrates consist of atypical lymphocytes, often extending into the deep dermis and subcutaneous fat. Adnexal invasion is frequently observed (242). Blistering, angiocentricity, angioinvasion, riming of adipocytes, and destruction of adnexal structures may be seen (240). Cells invariably demonstrate CD8^+^CD4^−^ phenotypes and usually express CD3, β-F1, and TIA-1. CD45RA is expressed in the majority of cases (239). T-cell clonality is usually demonstrated. Conventional therapies for CTCL are ineffective and multiagent chemotherapies have unsatisfactory outcomes (240). Hematopoietic stem cell transplantation is a reasonable treatment choice for PCAETCL (243).

## Author Contributions

The author confirms being the sole contributor of this work and approved it for publication.

## Conflict of Interest Statement

The author declares that the research was conducted in the absence of any commercial or financial relationships that could be construed as a potential conflict of interest.
